# A Study of Genomic Prediction of 12 Important Traits in the Domesticated Yak (*Bos grunniens*)

**DOI:** 10.3390/ani9110927

**Published:** 2019-11-07

**Authors:** Donghai Fu, Xiaoming Ma, Congjun Jia, Min Chu, Qinhui Lei, Zhiping Wen, Xiaoyun Wu, Jie Pei, Pengjia Bao, Xuezhi Ding, Xian Guo, Ping Yan, Chunnian Liang

**Affiliations:** 1 Animal Science Department, Lanzhou Institute of Husbandry and Pharmaceutical Sciences, Chinese Academy of Agricultural Science, Lanzhou 730050, Chinachumin@caas.cn (M.C.); wuxiaoyun@caas.cn (X.W.); peijie@caas.cn (J.P.); dingxuezhi@caas.cn (X.D.); guoxian@caas.cn (X.G.); 2 Key Laboratory for Yak Genetics, Breeding, and Reproduction Engineering of Gansu Province, Lanzhou 730050, China; 3 Gannan Provincial Institute of Animal Science, Gannan 747000, China; zhipinwen123@163.com

**Keywords:** domesticated yak (Bos grunniens), genomic prediction, growth performance, disease resistance, prediction accuracy

## Abstract

**Simple Summary:**

The domesticated yak is among the most important livestock species on the Qinghai-Tibet Plateau. Breeders have the task of developing varieties that provide growth performance and disease resistance. Traditional breeding processes rely on complete family pedigree information and large numbers of data records. However, there are inevitably records that are missing, including incomplete pedigrees and long-term data tracking, resulting in prolonged breeding cycles, reduced breeding efficiency, and the lack of economic benefit. Genome selection (GS), also known as whole genomic selection (WGS), can significantly reduce the selection cycle of quantitative traits and accelerate genetic progression while displaying appropriate prediction accuracy (PA). It combines a reference population and single nucleotide polymorphism (SNP) loci rather than pedigrees to estimate the effect of all SNPs. Then breeding values of target traits are predicted. The key for GS is genomic prediction (GP) and an assessment of PA.

**Abstract:**

The aim of this study was to explore the possibility of applying GP to important economic traits in the domesticated yak, thus providing theoretical support for its molecular breeding. A reference population was constructed consisting of 354 polled yaks, measuring four growth traits and eight hematological traits related to resistance to disease (involved in immune response and phagocytosis). The Illumina bovine HD 770k chip was used to obtain SNP information of all the individuals. With these genotypes and phenotypes, GBLUP, Bayes B and Bayes Cπ methods were used to predict genomic estimated breeding values (GEBV) and assess prediction capability. The correlation coefficient of the association of GEBV with estimated breeding value (EBV) was used as PA for each trait. The prediction accuracy varied from 0.043 to 0.281 for different traits. Each trait displayed similar PAs when using the three methods. Lymphocyte counts (LYM) exhibited the highest predictive accuracy (0.319) during all GP, while chest girth (CG) provided the lowest predictive accuracy (0.043). Our results showed moderate PA in most traits such as body length (0.212) and hematocrit (0.23). Those traits with lower PA could be improved by using SNP chips designed specifically for yak, a better optimized reference group structure, and more efficient statistical algorithms and tools.

## 1. Introduction

The polled yak is a newly cultivated breed that provides meat, milk, and other products to local people on the Qinghai-Tibet Plateau [1]. Compared with the common domesticated yak, they exhibit a hornless trait with better growth performance [2]. Due to the disappearance of horns, evolutionarily used for self-defense and in competition for a mate, the polled yak is suitable for large-scale production and resource management [3].

The majority of important economic traits of yaks are affected by multiple genes [4]. Conventional breeding depends mainly on an assessment of appearance, measurement of performance, pedigree, and assessment of phenotypes to estimate breeding values [5]. These methods have resulted in great progress in yak breeding in the past. However, the yak has a long generation interval with a low rate of reproduction. Furthermore, important economic traits are difficult to measure, which in any case are too costly, thus making improvements to livestock traits relatively slow [6].

In recent years, with the development of molecular genetics, molecular markers have been defined for marker-assisted selection (MAS) in the breeding of cattle to improve the accuracy of selection [7]. However, it has been found that the number of major effect genes affecting economic traits are limited [8]. In view of the poor performance of MAS in many economically-valuable animals, Meuwissen et al. proposed a genome-wide marker-assisted selection method in 2001, the whole genome selection method [8]. With the publication of the bovine genome-wide sequence, high-density, high-throughput commercial SNP chips were subsequently developed, resulting in improved genotyping efficiency at a reduced cost [9].

Genomic selection has been utilized on a large scale in major economic animals such as dairy cattle [10], pig [11], sheep [12] and beef cattle [13] since it was proposed by Meuwissen et al. [8]. The focus of genome selection is the prediction of individual GEBV and accuracy of genomic prediction in the experimental population. Common statistical methods, including genomic best linear unbiased prediction (GBLUP), Bayesian methods (Bayes A, Bayes B, Bayes Cπ, etc.), single step BLUP (ssBLUP), and other algorithms were considered for genomic selection and prediction [14]. In this study, three statistical methods (GBLUP, Bayes B, and Bayes Cπ) were selected for the prediction of the GEBV of four growth traits and eight hematological traits according to previous genome-wide association analysis (GWAS) results [15].

The relationship between relevant blood traits and the resistance of yak to disease has been discussed in earlier studies and therefore will not be repeated here. Production performance and hematological traits play vital roles in the process of intensive farming of the domesticated yak [16]. However, genomic selection in the yak population has not been utilized. Hence, the possibility of utilizing GP to select for four performance traits and eight disease resistance traits was explored in this study.

In this study, by selecting a reference population and obtaining SNP chip genotyping, the genomic prediction of a yak growth performance index and blood biochemical index was conducted to verify the application of GS in the yak population and to explore related optimization methods.

## 2. Materials and Methods

This study was approved by the Chinese Academy of Agricultural Sciences Laboratory Animal Ethics Review with accession number CAAS 2017-115. All procedures were in compliance with the guidelines for the care and use of experimental animals. The experiment station, Qinghai Datong Breeding Farm is located in Datong County, Xining City, Qinghai Province, China (36°52’ N, 101°23’ E), with an average elevation of 3450 m. Datong County has a plateau continental climate and an average annual temperature of 4.9 °C.

### 2.1. Experimental Population Selection

The animal population used in this study comprised of 354 polled yaks selected from the Datong breeding farm in Qinghai Province, China. They were all healthy female individuals born between March and May 2017. These yaks were scattered across three breeding farms and were grazed naturally.

### 2.2. Phenotype Data Acquisition and Processing

The phenotypic traits of the 18-month-old yak reference population were obtained in October 2018, including direct measurement of performance data, withers height, body length, body weight, chest girth (WH, BL, BW, CG) and eight hematological traits obtained from non-anticoagulated blood, namely, red blood cell count, hemoglobin, hematocrit, platelet count, lymphocyte count, medium white blood cell count, distribution of platelet width, and mean platelet volume (RBC, HGB, HCT, PLT, LYM, OTHR, PDW, and MPV), as described in a previous study [15]. Twelve phenotypic traits were tested for normality and those values falling more than 3 standard deviations from the mean were removed using SAS (version 9.2) (SAS Institute INC, North Carolina, NC, USA) [17].

### 2.3. SNP Chip Genotyping and Quality Control

Genomic DNA was extracted and genome-wide single nucleotide polymorphism (SNP) loci scanned using an Illumina Bovine HD 770K chip, as described in detail in a previous study [18]. PLINK software (version 1.90) (Purcell laboratory, Boston, MA, USA) was used to perform quality control [19]. All SNPs with minor allele frequencies <0.01, an SNP detection rate <0.05 and HWE <10^−6^ were removed. Those individuals with an individual detection rate <0.1 and Mendelian errors >5% were also removed. Only SNPs and individual yaks that satisfied the quality control requirements described above were selected for subsequent research and analysis.

### 2.4. Method of Calculation of GP

Methods of calculation of genome prediction are divided into two categories. One is the method of directly estimating breeding value, based on the genetic relationship matrix (G matrix) that predicts genomic breeding value [20]. The other is a method that indirectly estimates breeding value, by accumulating the effects of all SNPs in order to predict genomic estimated breeding values (GEBV) [21].

The technique used for directly estimating genomic breeding value is the genomic best linear unbiased prediction (GBLUP) method, based on the best linear unbiased prediction (BLUP) method [22]. Genome-wide SNPs are combined into a G-matrix instead of a pedigree matrix (A-matrix), which is substituted into a mixed model equation for solving, to finally estimate individual GEBVs. Additionally, the GBLUP effectively reduces the size of the estimation matrix and so reduces computational intensity [23]. The statistical model for GBLUP is as follows:

y = Xb + Zg + e(1)

where *y*, *X*, *b*, *Z*, *g*, and *e* are the phenotype vector, matrix relating the fixed effects to each animal, fixed effects vector, correlation matrix of random additive genetic effects, random additive genetic effects vector (that is, the individual genotype breeding value), and the residual vector, respectively. Prediction of GEBVs and the assessment of accuracy of GBLUP were conducted using the GAPIT function set [24] in R project software (www.r-project.org) [25].

In a previous study, a number of SNP loci associated with eight hematological traits were explored through GWAS [15]. Differing from the assumption of Bayes A, the assumption of Bayes B is that a small number of SNP loci have an effect but the majority of loci do not, across the whole genome [26]. The GEBV is calculated using the following formula:

(2) $$y\;=\;\mathrm{Xb}\;+\;\Sigma_{i=1}^{m}Z_{i\;}g_{i\;}+\;e$$

where *y* is a phenotypic vector, *X* is a fixed-effect correlation matrix, *b* is a fixed effect vector, $g_{i}$ is the effect value of i SNPs, *m* is the total number of SNP sites, and *e* is the random residual effect vector. *Z* is a haplotype correlation matrix of n × *m*, where n is the number of individuals. In fact, the number of quantitative trait loci (QTLs) affecting the target trait is also limited, consistent with the theoretical hypothesis of the Bayes B method [27].

Bayes Cπ is an improvement on the Bayes B method. In a Bayes A-based model, the mean of the inverse chi-square distribution can be determined by information such as additive genetic variance of the trait, thereby estimating the ratio π of the non-effector site [28]. Similar to Bayes B, Bayes Cπ assumes that some SNP loci have an effect and the ratio of their effect values is 1-π. The effects are described by a normal distribution and the effect variances are assumed to be the same. 

The π in the Bayes Cπ model is not a constant, but assumes that the prior distribution obeys a uniform distribution denoted as Uniform (0,1) and that its posterior distribution is a beta distribution [29,30]. In this study, Bayes B and Bayes Cπ models were used to calculate the GEBV using lme4 [31] and BGLR [32] packages in the R project. By adding all SNP locus effect values, the genomic breeding values of all individuals could be calculated. 

### 2.5. Evaluation of Genetic Parameters in Yak Population

In this study, the mrMLM package (version 3.1) (Nanjing Agricultural University, Nanjing, China) available in R project software was used to evaluate genetic parameters, including the additive genetic effect, the error effect and the fixed effect [33]. Different kinship was treated solely as a fixed effect because all individuals were female and of the same age. Heritability ($h^{2}$) was obtained by dividing the additive genetic variance by the total variance. The genetic parameters model is as follows:

Phenotype = additive effect + fixed effect + error effect(3)

### 2.6. Assessment of Accuracy of Various Models

In general, calculation of accuracy is required in order to evaluate the predictive capability in different models and different traits [34]. In this study, the correlation between phenotype y* corrected by the validation population and GEBV was divided by the square root of the heritability of the trait. Accuracy of the estimation of genomic breeding value for each method was evaluated as a parameter (r, Realized Accuracy) [35]. Accuracy r is calculated as follows:

(4) $$r_{\mathrm{GEBV}}\;=\;\frac{\mathrm{Cor}\;\left(y*,\;\mathrm{GEBV}\right)}{\sqrt{h^{2}}}$$

Five-fold cross validation was performed to assess the accuracy of GP in this study. All individuals were randomly divided into 5 groups on average, of which 4 were used as a training group to estimate parameters, and the other group used to test the group (validation group).

## 3. Results

### 3.1. Quality Control Results for Genotype Data

From the filtering process described above, the following quality control (QC) results were obtained, as shown in Table 1:

After filtering, the 96,087 SNP loci and 354 individuals remaining were used for GS.

### 3.2. Basic Statistics of Phenotype Data

All phenotype data were processed as described above, displaying a symmetric distribution, as detailed in Table 2.

### 3.3. Estimation of Genetic Parameters

By using the mrMLM package in the R project with exisiting data, all genetic parameters were estimated, as summarized in Table 3.

### 3.4. GP Results Using the Three Methods

In this study, GBLUP, Bayes B, and Bayes Cπ were selected as methods to predict the GEBVs of 12 target traits with processed data, including phenotype and genotype data. The accuracy of prediction of GBLUP, Bayes B, and Bayes Cπ is listed in Table 4.

**Figure 1:**
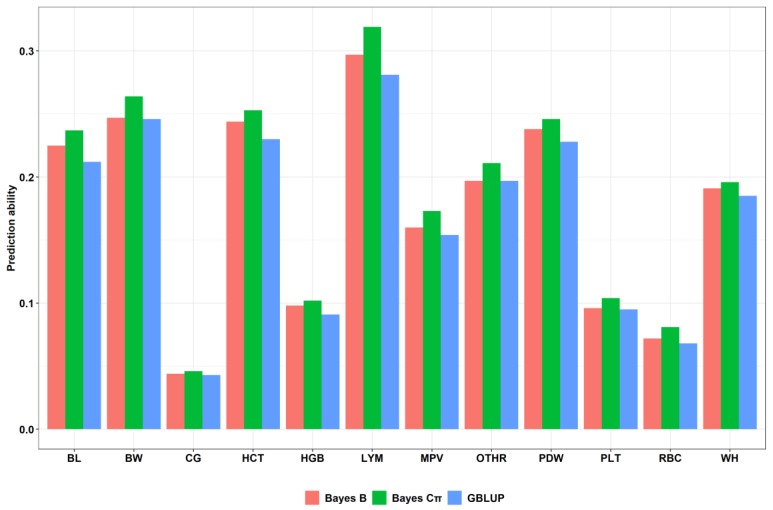
Accuracy of prediction of three methods for 12 target traits. Note: Colors represent the different methods. The abscissa represents different traits and methods. The ordinate is the corresponding prediction capability.

As can be seen from Table 4, prediction capability acquired from 5-fold cross validation differs among the 12 target traits. LYM had the highest prediction accuracy using Bayes Cπ (0.319) while CG had the lowest in GBLUP (0.043). Several traits, such as BW and HCT, displayed moderate predictive accuracy (0.154–0.319) while CG and RBC exhibited quite a poor predictive accuracy (<0.1), regardless of the method used. Interestingly, a slight improvement in predictive accuracy was found for HGB and PLT, reaching a moderate level (>0.1) when using Bayes Cπ. GBLUP, Bayes B, and Bayes Cπ showed similar prediction capability in any of the 12 traits (Table 4 and Figure 1). However, Bayes Cπ represented the method with the best prediction capability across the 12 target traits and should be selected as the optimal method (Figure 1).

## 4. Discussion

In this study, GBLUP, Bayes B, and Bayes Cπ methods were used for GS and assessment of PA in a yak population. A bovine HD chip was used to scan the yak genome and all SNPs were cleaned through a defined process [19]. As can be seen from Table 1, fewer genotyped SNPs (96,087) were obtained from the 777,962 contained in the Bovine HD chip, the proportion of SNPs removed being 87.64%. We re-examined the QC processes again and found that the total genotyping rate was 0.973008. A total of 44,585 SNPs was removed due to missing genotype data (>0.05) and 1001 SNPs removed due to the results of the Hardy–Weinberg exact test (<1 × e^−6^). In addition, 636,289 SNPs were removed due to the minor allele frequency that is less than 0.01 (<0.01) in the majority of all removed SNPs. Furthermore, all yaks had less than 10% missing genotype data so no one was removed. The fact that so many loci with a minor allele frequency of less than 0.01 were removed may be because of the use of bovine chips instead of a specific yak chip and that these SNP genotypes are rare in the entire population.

When estimating the effect of an SNP, it is necessary to combine observed phenotypes and genotypes. However, various errors may occur in actual situations. Using probability theory, the greater the number of experiments performed, the more frequently these errors occur [36]. Therefore, it is necessary to record more individual phenotypic information to reduce measurement error. In other words, larger numbers of reference groups result in a more accurate estimation of the effect of a marker. Meuwissen et al. concluded through simulation experiments that accuracy was greatest if animals per population was greater than 2000 for estimating the breeding value of a trait with a heritability of 0.3 [8]. According to Goddard et al., the lower the heritability of traits, the more animals are required, and traits with a heritability of 0.1 require more than 10,000 animals per sample to ensure an accuracy of 0.4 [37]. A study by Habier et al. demonstrated that the accuracy of GP decreases with increasing generations between the reference and validation populations [38]. In the present study, 12 phenotypic traits with medium heritability were studied in a reference population consisting of 354 polled yaks from 3 family lineages for genome prediction, with a maximum accuracy of 0.319. Accuracy of prediction is expected to be improved after expanding the size of the reference population and considering population structure.

The premise of GS is that it predicts quantitative traits from multiple linkage disequilibria (LD) for each quantitative trait locus (QTL), which is influenced by the number of SNPs in a gene chip. That is to say, the greater the number of markers, the higher the accuracy. Calus et al. believe that the larger the rate of linkage, the greater the accuracy [39]. That is, increased chip density and capacity can improve accuracy. Although a 770K high-density chip was used in this study, the number of SNPs after filtration decreased to 96K due to the species differences between the yak and cattle, which may result in some specific SNP loci in the yak population not being detected. However, the use of gene filling technology to increase the resolution of low-density chips into medium- or high-density chips can improve the accuracy of GPs while controlling costs [40]. 

For simulation studies of different species, Bayes methods have been demonstrated to be better than GBLUP. The results of this study confirm this conclusion. Sun et al. analyzed the simulation data from the 14th QTL-MAS Workshop, the results indicating that the accuracy of Bayes Cπ was approximately 6% higher than that of GBLUP [41,42]. Edriss et al. used 4429 Danish Holstein bulls and 50K chip data to estimate breeding values and the results also showed that Bayes Cπ was more accurate than GBLUP [43]. Niu. H et al. used a 770k high-density chip to predict slaughter traits of Chinese Simmental cattle, utilizing a 10-fold average of cross-validation as accuracy of prediction. The results indicated that Bayes Cπ had the greatest accuracy and GBLUP the lowest [44]. Lin. et al. conducted genome prediction analysis on simulated data of the backfat of pigs and came to the same conclusion [45]. In the studies of Edriss, Niu. H and Lin, Bayes Cπ showed the highest predictive capability despite the different species and traits they studied. The results of the studies described above are consistent with the present study.

## 5. Conclusions

The present study was the first time GP has been applied to the domesticated yak population, which assessed prediction accuracies of 12 important traits using GBLUP, Bayes B, and Bayes Cπ. The three methods presented approximate prediction capability. Several traits such as BL, WH, and BW exhibited moderate prediction accuracy (>0.1), demonstrating that it is possible to apply GP/GS in yak breeding. Unfortunately, GP/GS demonstrates a tiny effect on CG and RBC, with low accuracy (<0.1).

## Figures and Tables

**Table 1:** Statistical summary of all single nucleotide polymorphism (SNP) loci and individuals before and after quality control (QC).

Description	Count
Original SNPs	777,962
Original Individuals	354
SNPs left after QC	96,087
Individuals after QC	354

**Table 2:** Summary statistics of the 12 phenotypic traits.

Traits	Abbreviation	Values ^1^	Counts ^2^
Body length (cm)	BL	101.613 ± 5.683±	320
Body weight (kg)	BW	122.39 ± 12.397	259
Chest girth (cm)	CG	137.803 ± 8.784	315
Withers height (cm)	WH	101.765 ± 5.816	319
Red blood cell count (10^12^/L)	RBC	10.124 ± 1.083	308
Hemoglobin (g/L)	HGB	137.136 ± 16.794	310
Hematocrit (%)	HCT	0.430 ± 0.052	306
Platelet count (10^9^/L)	PLT	326.193 ± 169.875	311
Lymphocyte count (10^9^/L)	LYM	4.475 ± 1.5478	273
Medium white blood cell count (10^9^/L)	OTHR	4.550 ± 1.426	267
Platelet distribution width (%)	PDW	8.776 ± 1.310	169
Mean platelet volume (fl)	MPV	7.146 ± 0.624	171

^1^: Values are shown in mean ± standard error of the mean. ^2^: The number of recorded individuals.

**Table 3:** Summary of the genetic parameters of different traits.

Trait	F + E ^1^	Phenotype ^2^	Additive ^3^	$\mathrm{Heritability}\;(h^{2}){\;}^{4}$
BL	21.002	33.141	12.140	0.366
BW	83.011	168.709	85.698	0.508
WH	15.434	37.590	22.156	0.589
CG	66.251	101.141	34.890	0.345
RBC	0.909	1.182	0.273	0.231
HGB	175.259	279.663	104.404	0.373
HCT	0.002	0.003	0.001	0.444
PLT	15,282.669	28,121.015	12,838.346	0.457
LYM	1.147	2.140	0.993	0.464
OTHR	1.099	2.038	0.939	0.461
PDW	0.737	1.576	0.839	0.533
MPV	0.149	0.383	0.233	0.610

^1^: Including fixed effect variance and error effect variance; ^2^: Total phenotypic effect variance; ^3^: Addictive effect variance; ^4^: The ratio of the additive effect variance to the total phenotypic variance.

**Table 4:** Statistical summary of the accuracy of prediction of the 12 target traits.

Trait	Prediction Accuracy Using Individual SNPs		
	GBLUP ^1^	Bayes B ^2^	Bayes Cπ ^3^
BL	0.212	0.225	0.237
BW	0.246	0.247	0.264
WH	0.185	0.191	0.196
CG	0.043	0.044	0.046
RBC	0.068	0.072	0.081
HGB	0.091	0.098	0.102
HCT	0.23	0.244	0.253
PLT	0.095	0.096	0.104
LYM	0.281	0.297	0.319
OTHR	0.197	0.197	0.205
PDW	0.228	0.238	0.246
MPV	0.154	0.16	0.173

^1^: Genomic best linear unbiased prediction; ^2^: Bayesian method B; ^3^: Bayesian method Cπ.
